# Historical Perspectives on the Epidemiology of Human Chagas Disease in Texas and Recommendations for Enhanced Understanding of Clinical Chagas Disease in the Southern United States

**DOI:** 10.1371/journal.pntd.0003981

**Published:** 2015-11-05

**Authors:** Melissa N. Garcia, Laila Woc-Colburn, David Aguilar, Peter J. Hotez, Kristy O. Murray

**Affiliations:** 1 Department of Pediatrics, National School of Tropical Medicine, Baylor College of Medicine and Texas Children’s Hospital, Houston, Texas, United States of America; 2 Department of Medicine, Baylor College of Medicine, Houston, Texas, United States of America; Universidad Centroamericana, NICARAGUA

## Abstract

Chagas disease (*Trypanosoma cruzi* infection) has recently been identified as an important neglected tropical disease in the United States. Anecdotally referred to as a “silent killer,” it leads to the development of potentially fatal cardiac disease in approximately 30% of those infected. In an attempt to better understand the potential of Chagas disease as a significant underlying cause of morbidity in Texas, we performed a historical literature review to assess disease burden. Human reports of triatomine bites and disease exposure were found to be prevalent in Texas. Despite current beliefs that Chagas disease is a recently emerging disease, we report historical references dating as far back as 1935. Both imported cases and autochthonous transmission contribute to the historical disease burden in Texas. We end by discussing the current knowledge gaps, and recommend priorities for advancing further epidemiologic studies and their policy implications.

## Introduction

Carlos Chagas first described the protozoan parasite *Trypanosoma cruzi* after isolation of the organism from the blood of a Brazilian patient in 1909 [1]. Research since then has allowed us to understand that natural transmission of *T*. *cruzi* occurs between humans and an insect vector, particularly *Triatoma* species from the Reduviidae family [2]. The vectors are nocturnal feeders, at which time they concurrently release *T*. *cruzi*-infected feces on the skin. Parasites enter the body through breaks in the skin or through mucous membranes. Other less common routes of transmission include congenital transmission, ingestion of contaminated food or beverage products, and transfusion of contaminated blood, tissue, or organs [2].

Acute disease normally presents as either facial edema and/or non-specific flu-like illness, but can be more severe in immunosuppressed patients [3]. The chronic phase of infection with *T*. *cruzi* is lifelong, with approximately 30% of infected persons developing cardiac complications and Chagasic cardiomyopathy characterized by arrhythmias, ventricular aneurysms, decreased ejection fraction, and sudden cardiac death [4]. Clinical manifestations develop over the course of 10 to 30 years and are clinically apparent in later stages of disease [2]. Early diagnosis and treatment is important for the prevention of irreversible cardiac function impairment.

An estimated 7.5 to 10 million persons are infected with Chagas disease worldwide [5, 6]. The majority of infections occur in endemic countries throughout Central and South America. While the United States is generally considered a non-endemic area, recent publications have suggested that Chagas disease is emerging as a significant public health concern [7]. A large number of manuscripts from Texas, in particular, have recently been published on *T*. *cruzi* infection in vectors, mammalian reservoirs, and humans. Texas’ neighboring states and bordering Mexico states have also reported a number of Chagas disease cases and serious disease burden [2, 8–15]. Despite the numerous publications related to Chagas disease in the southern US and northern regions of Mexico, very little is known about the disease burden from imported and locally acquired *T*. *cruzi* infection.

Due to its proximity to Latin America, Texas’ population has a high proportion of immigrants. It is reasonable to conclude that imported cases from highly endemic areas in Latin America would likely occur in Texas. Additionally, we argue that local transmission has been occurring in the state for the past seven decades, as evidenced by published reports supporting *T*. *cruzi*-positive vectors, mammalian reservoirs (including dogs), and human cases. There is concern that Chagas disease might be undiagnosed in the US as a result of documented low physician awareness [16]. Here, we focus on human-related published reports to better describe historical evidence of both autochthonous *T*. *cruzi* transmission and imported *T*. *cruzi* infection among current Texas residents. Finally, we discuss the current gaps in scientific knowledge, and recommend steps to be taken for enhanced understanding of *T*. *cruzi* as a significant underlying cause of morbidity in the state of Texas and adjoining states.

## History of Clinical Human Chagas Disease in Texas

### 20^th^ Century Studies

The first documentation of triatomine vectors in relation to household infestation were reported by Wood and Packchanian in the 1930s [17, 18]. These two authors focused on the interactions of triatomine species with humans at a time when an understanding of Chagas disease was still in its infancy. Wood interviewed seven residents in the southwestern part of the state, whose anecdotal accounts of triatomine home infestations resulting in multiple nightly bites date back to 1935 [18]. One particular account stated, “*Triatoma* were so abundant that she could not prevent them from feeding on her while asleep.” Another interviewee noted “20 insects in her bed on one occasion.” Triatomine vectors and animals collected from this area tested positive for *T*. *cruzi* parasite; however, none of the residents interviewed were reportedly tested.

Packchanian’s preliminary study assessing *Triatoma gerstaeckeri* infection in October 1937 to September 1938 also included interviews with local residents [17]. In an area where 92% of collected vectors were found to be positive for *T*. *cruzi* infection, the author queried residents about history of triatomine bites. More than 500 people from the Three Rivers, Texas area reported bite histories. One woman, in particular, stated that “her family had killed 300 or more ‘blood suckers’ every night for 6 weeks” [17]. The majority of the *T*. *cruzi*-infected *T*. *gerstaeckeri* collected had come from in and around homes. In an attempt to understand the infectivity of the Texas *T*. *cruzi* strain to cause morbidity in humans, Packchanian ocularly infected a 24-year-old African-American male and successfully demonstrated pathogenicity of this local circulating strain [19].

Wood and Packchanian’s preliminary findings of *T*. *cruzi* infection in vectors and the potential for human transmission caught the attention of the Texas State Department of Health (now known as Texas Department of State Health Services). The state department conducted a large seroprevalence study in southern Texas in 1942 [20]. A total of 1,909 human serum samples were tested by complement-fixation tests, resulting in a low infection prevalence of 0.05% (1/1,909) [20]. The lone positive sample was from an 8-year-old boy living in Uvalde County, where *T*. *cruzi*-positive triatomines were captured in the vicinity [20]. Following these studies, the first physician awareness and education article was published in 1947, summarizing the clinical manifestations of Chagas disease in humans, describing contemporary risk factors for acquiring infection, and commenting that human disease was likely occurring despite the current lack of detection [21].

Several years passed before another report emerged from the state. In 1950, a commentary to an article discussing severe reactions from insect stings was published. In this commentary, a physician from Temple, Texas, mentioned that one of his staff nurses presented with multiple episodes of facial, throat, and ankle edema of unknown origin occurring at night [22]. The edema was severe enough that adrenalin and antihistamines were given on three occasions. Eventually, they identified the cause as Chagas disease after catching a *Triatoma*. Unfortunately, the nurse’s and the triatomine’s *T*. *cruzi* infection status were never reported.

Not soon after, in 1955, Woody and Woody documented the first autochthonous *T*. *cruzi* infection in a 10-month-old girl from Corpus Christi, Texas, who had a relatively unremarkable course of infection [23]. The family reported having “blood-sucker” infestations of the home, but the girl’s history of insect bites was unknown. Several months later, a second pediatric case was reported from a Houston hospital [24]. Unfortunately, the exact details of this second case were never fully published; however, it was noted that trypanosomes were detected in the cerebrospinal fluid of the 6-month-old boy after a long hospitalization [24, 25].

Concurrently, dermatologists in Fort Worth, Texas, wrote a review of acute Chagas disease clinical presentation after noting a high proportion of patients presenting with lesions caused by the bite of a triatomine insect. Over a 2-year time span, 45 patients presented with triatomine bites, and they were all able to bring the clinicians triatomines found in their beds for identification [26]. Notably, the authors commented that “our patients came from all parts of the city, from all types of dwellings, and from all economic levels” [26]. Regrettably, there is no record of blood samples from these 45 patients or their collected triatomines being tested for *T*. *cruzi* infection. By the late 1950s, a Galveston, Texas, researcher became interested in understanding the clinical practices of state physicians in regards to treatment of various insect stings or bites. From his investigation of 1,905 patients, he discovered seven patients who had triatomine bug bites from 1955 to 1959 that required medical attention [27]. Triatomine bite reactions ranged from general fever to anaphylactic shock. Yet again, their *T*. *cruzi* infection status was unknown.

Following the index autochthonous patient, the same physicians (Woody and Woody) performed two serologic studies of potential high-risk groups in the 1960s: (1) children that presented to a Corpus Christi hospital that serves indigent populations, and (2) persons bitten by a triatomine vector. In the pediatric population (which included testing of four adult family members), a seroprevalence of 1.8% (nine out of 500) was found. Positive children (n = 7) ranged in age from 4.5 to 15 years, adult family members (n = 2) ranged 45 to 53 years, and all were Latin-American [28]. All seven infected children reported potential exposures to vectors, and *T*. *cruzi*-infected triatomine vectors were collected from 57% (four out of seven) of these children’s homes. Only four family members of these infected children were tested, of which two adults (50%) were positive for infection.

From the insect-bite exposure population, 2.5% (three out of 117) tested positive for *T*. *cruzi* infection [29]. The duration of bite history to date of testing ranged from 3 to 10 years. Ages of the three positive persons varied greatly (5, 42, and 72 years old). None of the three had recorded medical histories consistent with *T*. *cruzi* infection. A third seroprevalence study was performed by the US Communicable Disease Center, now known as US Centers for Disease Control and Prevention (CDC), and the USAF Epidemiological Laboratory at Lackland Air Force Base in San Antonio, Texas. In July 1964, a USAF sergeant brought in two triatomines that had been biting himself and his family on numerous occasions [30]. Following this incident, a seroprevalence study was conducted of residents living near where the bugs had been collected (20 miles northeast of San Antonio, Texas). One out of 108 residents tested was positive for *T*. *cruzi* antibodies [30, 31]. The 63-year-old male who tested positive had no clinically compatible symptoms. Of these 108 persons tested, 48 reported a history of triatomine bites. Interestingly, however, the *T*. *cruzi*-positive person did not report a history of triatomine bites.

The 1970s were quiet with regards to Chagas disease studies in Texas. In the late 1970s to early 1980s, one seroprevalence study in humans was conducted along the Texas–Mexico border. Researchers in Hidalgo and Cameron Counties (eastern portion of the Texas–Mexico border) found *T*. *cruzi* prevalence rates of 2.4% (12 out of 500) in humans [32]. The source of infection was not investigated among the 12 infected persons; however, all were long-time residents of the Rio Grande Valley. One of the 12 infected had previously been diagnosed with idiopathic cardiomyopathy.

Between the 1980s and the 1990s, three unique human case reports and one large blood-borne transmission serologic study were published. The first case report detailed the findings from a fatal case of cardiogenic shock in a 59-year-old female suspected to be caused by a *T*. *cruzi*-infected blood transfusion [33]. Next, a fatal case of acute myocarditis that was locally acquired in a 7-month-old male was reported [34]. Of note, the infant in the case report was diagnosed at the same hospital as the first reported autochthonous case [23]. The last case report was an imported Chagas cardiac case in a 55-year-old female who presented with multiple left ventricular aneurysms in the anterobasal, anterior, and inferior aspects of the left ventricle [35].

Out of mounting concern for the possibility of *T*. *cruzi* blood transfusion in the US, the American Red Cross performed a multi-site serologic blood transfusion study. Between 1988 and 1991, two hospital sites from Houston, Texas, recruited a total of 7,738 patients undergoing cardiac surgeries and requiring blood transfusions [36]. Five patients (0.06%) were confirmed positive for *T*. *cruzi* infection. The authors concluded that these patients had acquired infection prior to their transplantation and that no blood-transfusion–associated *T*. *cruzi* infection had occurred. Four patients were Hispanic and had been born in *T*. *cruzi*-endemic countries; however, the other positive patient was a non-Hispanic who was a native of Texas. Further evaluation to identify the source of infection was never performed.

### 21st Century Studies

The new millennium brought a renewal of interest and publications on a variety of entomological, environmental, mathematic modeling, and clinical aspects of *T*. *cruzi* infection in Texas. The studies focused on human infection in the 2000s were mostly comprised of seroprevalence and case reports of unusual disease presentation. Interestingly, a bioarchaeological study in Del Rio, Texas, implicated the first possible case of Chagasic megacolon in the area, dating back 1,150 years [37]. Another unique case report discussed a Dallas resident who presented with an unusual presentation of acute myocardial infarction as a result of his previously undiagnosed Chagas cardiac disease [38]. Fortunately, his cardiologists were able to properly recognize and manage his disease in a timely manner, resulting in improved health outcomes.

Since 2000, two case reports of Chagas disease in immunocompromised patients residing in Texas have been published. The first patient was a Honduran-born 29-year-old man who presented with HIV and acute congestive heart failure [39]. He died on the second day of hospitalization and was not diagnosed with Chagas disease until post-mortem examination revealed *T*. *cruzi* amastigote nests in the myocardium. The second patient was a 49-year-old Honduran-born woman who presented with meningoencephalitis and co-infection of HIV [40]. Upon diagnosis of *T*. *cruzi* in the cerebrospinal fluid on the fifth day of her hospitalization, the patient began a course of benznidazole. This patient is the first reported case of central nervous system Chagas reactivation in an AIDS patient that was successfully treated in the US. The contrasting outcomes of these two case reports show the importance of early diagnosis and treatment, particularly among immunocompromised patients.

Two important congenital risk studies were performed in Houston, Texas, over a 20-year duration. Seroprevalence testing of mothers from 1993 to 1996 revealed that 0.3% (11 out of 3,765) of pregnant women were *T*. *cruzi*-positive [41]. Of note, two out of the 11 positive mothers were non-Hispanic, with the rest being of Hispanic ethnicity. No additional information was available, as this was a convenience sampling of previously collected samples. Out of concern for congenital transmission in this population, the seroprevalence study was repeated from 2011 to 2012. A consistent perinatal infection rate of 0.25% (ten out of 4000) was found in expecting mothers [42]. Eight of the ten mothers were available for additional interviews. All eight mothers interviewed were from rural areas of Latin America and none had ever heard of Chagas. Six infants were available for repeat testing, and none had serologic evidence of *T*. *cruzi* infection.

The recent onset of blood donor screening for *T*. *cruzi* antibodies has provided a wealth of information in regards to understanding disease burden across the state. A study done in Waco, Texas, found a prevalence of 0.01% (three out of 23,021) of allogenic blood donations positive, with two of the three donors having given multiple donations prior to the implementation of screening [43]. Two of the donors from this screening study were born in Texas. Another investigation of banked sera samples from Hispanic-surnamed individuals in Dallas, Texas, revealed 0.4% (one out of 274) infection prevalence [44]. Similarly, a study in El Paso, Texas, found a prevalence of 0.03% (three out of 10,192) among blood donors [45]. A follow-up study from the same blood donor organization 5 years later revealed a prevalence of 0.01% (12 out of 93,009) in El Paso, Texas, 0.01% (two out of 19,811) in McAllen, Texas, and 0.001% (one out of 66,720) in Lubbock, Texas [46]. Over the first 5 years of routine blood donor screening in Texas, confirmed positive donors have come from 109 different ZIP codes [47]. These studies suggest geographic variance in infection prevalence, likely due to environmental conditions required to support vector habitat.

Despite the numerous publications since 2000 regarding human *T*. *cruzi* infection in Texas, none had specifically assessed locally acquired infection in a larger setting than individual case reports. Additionally, no studies to date had assessed cardiac manifestations of disease in positive residents. With the advent of blood donor screening in 2007, a representative sampling of *T*. *cruzi*-positive donors was available for such assessments. By combining seroprevalence data from multiple blood banks, we were able to identify a statewide prevalence of one per 6,500 Texans [47]. Upon enrollment of a pilot population of presumably healthy blood donors, we were surprised to discover that 41% had evidence of cardiac disease [48]. Of most concern is that the majority of our patients with a known Chagas disease diagnosis were not diagnosed by their physicians, likely due to a lack of physician knowledge [48]. This lack of connection between knowledge of Chagas disease and actual diagnosis of a patient is likely influenced by the high proportion of blood donors not having a history of travel to an endemic country. We unearthed a geographic clustering of autochthonous cases that had never been previously identified in the US [49]. Lastly, we identified novel risk factors for autochthonous infection related to hunting behaviors [50].

## Knowledge Gaps and Future Recommendations

**Fig 1:**
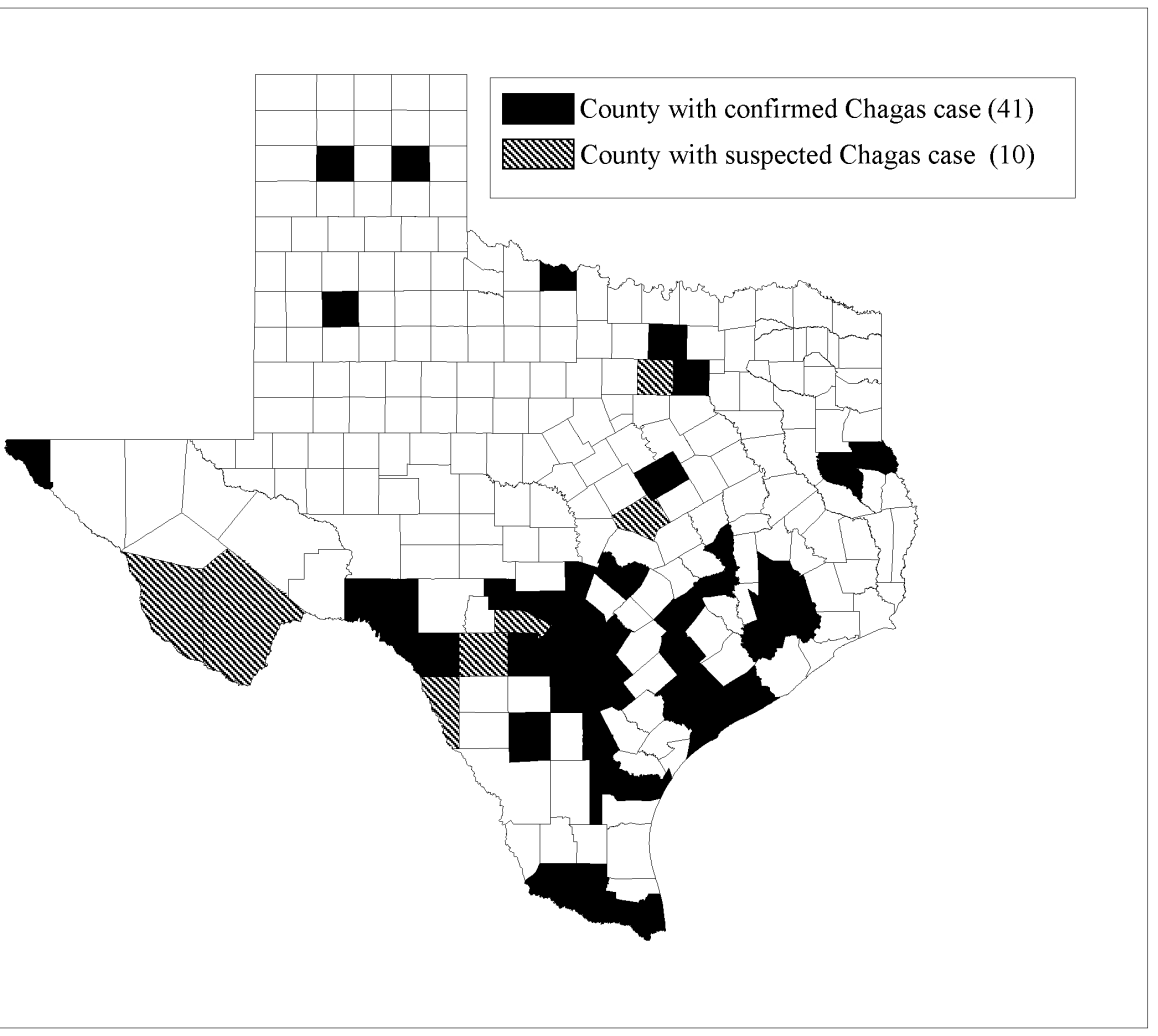
Texas counties of confirmed and/or suspected Chagas infection: publications from 1935–2015.

*T*. *cruzi* exposure from infected vectors to Texas residents dates back to 1935. The scientific literature supports the probability of frequent autochthonous infection in humans, as evidenced by compounding studies demonstrating established transmission cycles that have been occurring for more than half a century (Fig 1) (Table 1). It is imperative that we continue to identify which populations are at highest risk for infection. Texas is just one of 23 states with *T*. *cruzi*-infected vectors [2]. Multiple publications have highlighted the southern US as an area where autochthonous infection occurs, especially due to the high proportion of impoverished residents living in substandard housing infested by triatomines [2, 7, 51–57]. Large-scale screening of these states’ long-time residents would help elucidate the underlying disease burden attributable to *T*. *cruzi* infection. While it is necessary to understand prevalence of infection, it is also important to identify incident cases of infection. Our current limitation of antibody-based diagnostic screening prohibits us from identifying new incident cases. Repeat screening of blood donors and other high-risk populations would help to distinguish the rate at which transmission to humans is occurring on an annual basis.

**Table 1:** Evidence table containing all published reports of human-related Chagas activity in Texas.

Year*	Location	Number of subjects with vector bite history	Number of subjects testing positive for *T*. *cruzi* infection**	Additional comments	Reference
1935 to 1939	Chisos Mountains, TX; Shulma, TX; Bandera, TX; Quemado Valley, TX; El Paso, TX; Sullivan Mine, TX	Seven accounts of triatomine bite histories		“Miss B: *Triatoma* were so abundant that she *could not prevent* them from feeding on her while asleep; Mrs. O: took 20 insects from her bed on one occasion”	[18]
October 1937 and September 1938	Three Rivers, TX	500+ residents bitten in an area where 92% of *Triatoma gerstaeckeri* collected were *T*. *cruzi-*positive		Housewife stated they had killed “300 more bloodsuckers every night for 6 weeks”	[17]
December 1940	Three Rivers, TX		One man intentionally inoculated with *T*. *cruzi* parasite	“Material introduced into the left eye of an adult, male negro”; infection was confirmed from the patient’s blood 21 days post-exposure	[19]
1942	Uvalde County, TX		0.05% (one out of 1,909) infection prevalence	One positive case was an 8-year-old from Uvalde County, TX; very few details of his case	[20]
1950	Temple, TX	One nurse had nightly bite histories resulting in severe edema described in local physician’s *Abstract of Discussion*		Nurse had 3–4 episodes of facial, throat, and ankle edema that required pharmaceutical treatments on three occasions; a *Triatoma* was caught	[22]
July–August 1955	Corpus Christi, TX		First confirmed autochthonous case in the US	10-month-old girl presenting with fever, listlessness, and slight periorbital edema; overall unremarkable illness and recovery; father reported “blood-sucker” infestation in the home and mammalian hosts near residence	[23]
June–July 1955	Bryan, TX		Second confirmed autochthonous case in the US	2- to 3-week-old boy became ill, requiring hospitalization; trypanosomes detected in cerebrospinal fluid around 6 months of age; exact details never published.	[24, 25]
June 1955	Ft Worth, TX	45 patients with *Triatoma sanguisuga* bite lesions		Dermatologists wrote a triatomine bite review after having 45 patients present with Triatoma bite lesions and were able to bring in the insects from “in or about their beds” for identification	[26]
1955–1959	Entire state	Out of 1,905 patients treated for insect bites, seven (0.37%) patients treated for triatomine bites		Retrospective questionnaire of 124 TX physicians; seven patients treated for triatomine bites; five out of seven had severe systemic reactions; two out of seven had anaphylactic shock; one out of seven had fever; two out of seven had lymphadenitis	[27]
1961	Corpus Christi, TX		Nine out of 500 (1.8%) residents tested *T*. *cruzi* positive	Pediatric screening study found seven out of 496 children and two out of four adults *T*. *cruzi* positive; one out of nine patients (53-year-old male) had ECG abnormality; three out of nine had elevated serum gamma globulin levels; one out of nine had hepatomegaly and adenitis	[28]
1965	Corpus Christi, TX		Three out of 117 (2.5%) residents tested *T*. *cruzi* positive	None of the three had medical history of *T*. *cruzi* infection; Last bite ranged from 3 to 10 years prior to date of testing; Ages of cases were 5, 42, and 72 years old	[29]
1964	San Antonio, TX		One out of 108 (0.9%) persons tested *T*. *cruzi* positive	63-year-old male tested positive, but did not have evidence of clinically compatible disease	[30, 31]
1977–1978	Harlingen, TX		Twelve out of 500 (2.4%) persons tested *T*. *cruzi* positive	All 12 were longtime residents of the Rio Grande Valley; one out of 12 had unexplained cardiomyopathy; four out of 12 were confirmed by CDC testing	[32]
May 1989	Houston, TX		Fatal case report of a 59-year-old woman from a suspected *T*. *cruzi* blood transfusion	Longtime resident of Houston with no significant history of travel; undergoing radiation and chemotherapy for colon cancer when she developed severe pancytopenia and other clinical manifestations; patient died within 36 hours post-diagnosis of *T*. *cruzi* infection from fatal cardiogenic shock	[33]
July 1983	Corpus Christi, TX		Fatal case report of a 7-month-old boy with autochthonous vector-borne *T*. *cruzi* infection	Previously healthy infant hospitalized with acute myocarditis; *T*. *cruzi* diagnosis was made 7 years later by PCR testing of fixed cardiac tissue	[34]
1997	Austin, TX		Case report: an unusual presentation of multiple ventricular aneurysms	55-year-old woman with previous *T*. *cruzi* diagnosis made while living in South America; Case presented to Austin, TX cardiologist with three aneurysmatic dilations in the left ventricle	[35]
1987–1991	Houston, TX		Five out of 7,738 (0.06%) patients undergoing cardiac surgical procedures tested positive for *T*. *cruzi*	Cross-sectional study testing serum collected pre- and post-cardiac operation for *T*. *cruzi* antibodies; One of the five positive patients was suspected locally acquired case; All five were noted to have acquired infection prior to surgery; No evidence of blood transfusion as the route of T. cruzi infection.	[36]
853 BC	Del Rio, TX	Suspected case of Chagas disease in a recovered mummy		Mummy that died approximately 1,150 years ago had gross pathology implicating the potential for Chagas-related megacolon	[37]
2004	Dallas, TX		Case report: an unusual presentation of Chagasic cardiac disease	20-year Dallas, TX resident presented with acute myocardial infarction, focal left ventricular akinesis, and normal coronary arteries; patient was born and lived in Southern Mexico for 50 years before living in the US.	[38]
2004	Dallas, TX		Case report: Reactivation of Chagas cardiac disease in an AIDS patient	29-year-old male who was Honduras-born but had lived in the US for the preceding 5 years; *T*. *cruzi* infection reactivated in an AIDS patient which manifested as fatal acute congestive heart failure	[39]
2014	Houston, TX		Case report: Successful treatment of Chagas CNS reactivation in an AIDS patient	49-year-old female, Honduras-born; *T*. *cruzi* infection reactivated in an AIDS patient which manifested as meningoencephalitis	[40]
1993–1996	Houston, TX		Eleven out of 3,765 (0.3%) pregnant were confirmed positive for *T*. *cruzi* infection	Two out of the 11 sera were from non-Hispanic pregnant women, with the rest from Hispanic pregnant women; Age of infected mothers ranged from 18 to 33 years old	[41]
March 2011–April 2012	Houston, TX		Ten out of 4,000 (0.25%) pregnant women confirmed positive for *T*. *cruzi* infection	*T*. *cruzi*-positive mothers interviewed were all born in endemic countries; no evidence of congenital transmission	[42]
June 1996–April 1997	Waco, TX		Three sera out of 23,021 (0.01%) total blood donations were *T*. *cruzi* positive	These three sera were from three different donors; two of the three donors were born in the US, had never traveled outside the US, and had multiple generations being born in the US	[43]
November 2008–May 2009	Dallas, TX		One out of 274 (0.4%) tested positive for *T*. *cruzi* infection	Population was restricted to Hispanic-surnamed patients with evidence of Latin American immigration	[44]
2006	El Paso, TX		Three out of 10,189 (0.03%) total blood donations were *T*. *cruzi* positive	No additional demographic or clinical information was available; Two of the three positive samples were believed to be from the same donor	[45]
January 2007–December 2009	El Paso, TX; McAllen, TX; Lubbock, TX		Fifteen out of 179,540 (0.01%) blood donors confirmed positive for *T*. *cruzi* infection	Geographic variances ranged from 0.01% (12/93,009) in El Paso, 0.01% (2/19,811) in McAllen, TX, and 0.001% (1/66,720) in Lubbock, TX	[46]
January 2008–December 2012	Entire state		Of 907,398 blood donors, 140 (0.02%) confirmed positive for *T*. *cruzi* infection	Prevalence increased with age and was more likely among Hispanics; blood donors testing positive were significantly more likely to come from impoverished ZIP codes	[47]
2013–2014	Houston, TX		Prospective cohort analysis of 17 blood donors with confirmed T. cruzi infection	41% (seven out of 17) had evidence of Chagas-related ECG abnormalities; 36% (six out of 17) had evidence of locally acquired infection	[48, 49]

*Year of published report is listed when the article did not specifically list collection date(s) in the methods section

**Exact definition of *T*. *cruzi*-positive testing has changed over time, as a result of diagnostic modalities improving as technology naturally enhances with time

Our studies have revealed a unique group at risk for Chagas disease. Hunters have been implemented as a potential high-risk group for transmission due to their increased exposure in a sylvatic setting [50]. Hunters spend an average of 4.6 times more hours outside than the average US citizen [58, 59], possibly putting them in extended contact with vector habitats. A survey of Texas hunters revealed multiple exposures for *T*. *cruzi* transmission, including regular sightings of the vector, sleeping in infested housing structures, using hunting stands that might place them in a more intimate contact with vector nests, and not wearing gloves during skinning procedures to protect from possible blood-borne transmission [60]. In fact, the potential for transmission to hunters during the skinning process was first commented upon in a report from 1961 [25]. A recent case report highlighted these culminating risk factors in the case of an autochthonous infection in a 59-year-old Texas resident [49]. With natural infection being reported in multiple vector species and mammalian hosts across 28 states [2], it is imperative that we further understand the exact mechanisms for hunter-related *T*. *cruzi* transmission risk.

In addition to autochthonous infection, there are other populations of public health importance that should be investigated for disease burden. Indigent immigrant populations residing in the US are at risk for not accessing medical care and consequently remaining undiagnosed and untreated. With a potential 300,000 *T*. *cruzi*-infected Hispanic immigrants living in the US [61], we should increase our efforts to provide screening and care to this particular population, and educate physicians who serve these communities. The heightened need for this policy action was highlighted by a recent request for funding announcement initiated by the US Centers for Disease Control to fund proposals aiming to increase physician awareness of Chagas disease and other important neglected parasitic infections.

Persons with idiopathic cardiomyopathy and/or heart failure are another important group that should be targeted for public health intervention. A recent study found a 13% point prevalence of *T*. *cruzi* infection among New York City immigrants presenting with idiopathic cardiomyopathy [62]. Similarly, a study in Brazil found *T*. *cruzi* infection in 20% of those with heart failure [63]. This is particularly alarming because Chagas-related heart failure patients have a 4-fold higher mortality than traditional heart failure patients [64]. In addition to heart failure and sudden cardiac death, Chagas cardiac patients have an increased incidence of stroke and other cerebrovascular events [65, 66].

Lastly, pregnant women are an important high-risk group that should be considered for screening. Two studies performed in the same geographic area over a 20-year time period found a continuous substantial burden of *T*. *cruzi* infection among pregnant women in Texas [41, 67]. As of 2012, we now have confirmed congenital transmission that has occurred in the US [68]. This case report highlights the growing risk for congenital transmission. Screening of pregnant women for infection can greatly increase the infant’s chance of survival and decrease morbidity if disease is properly diagnosed and treatment is initiated early to both parties [69]. While there have been few studies assessing disease burden in the US, there is a growing consensus among the medical community that this is an important population for enhanced disease surveillance.

As more surveillance and seroprevalence studies of high-risk groups are conducted, we will be better able to assess the reality of risk as well as educate physicians on disease burden in our communities. It has been documented that physician awareness of this important infectious disease is low; however, what is particularly alarming is that only 11% of positive donors or their physicians seek treatment for disease from the CDC [16]. This finding was supported by our study, where only 25% of positive donors received additional follow-up care for their disease. Anecdotally, several of our study participants reported that their physicians told them they were “false positives” and that it was “impossible” for them to be infected. These troublesome facts indicate an urgency for increased awareness and education among physicians and clinicians who have regular contact with at-risk populations.

We would like to encourage the development of educational materials and initiatives for not only cardiologists and infectious disease specialists but also for primary care physicians, travel medicine clinicians, pediatricians, gastrointestinal internists, and obstetrician-gynecologists. Furthermore, only 47% of obstetrician-gynecologists had ever heard of Chagas disease [16]. Of those that were familiar with the disease, 68% reported not being up-to-date on their disease knowledge [16, 70]. Increasing awareness of these physician groups can improve patient prognosis when the clinician regularly considers diagnosis in their differentials.

Policy changes and increased funding of Chagas-related research is the next indispensable step in the prevention of *T*. *cruzi* infection. Better understanding of who is at highest risk for transmission is required so we can develop appropriate public health interventions. At our current juncture, only four states require Chagas disease to be a mandatory reportable condition. If all 23 states with a known infected vector would implement such policies, we could increase our knowledge base; but even then, making a disease reportable by itself has limitations. Ultimately, there is a need for programs of active surveillance in Texas and the southern US. Additional studies of vector and mammalian reservoir testing could identify geographic areas with high autochthonous transmission risk. Moreover, active screening of heart failure patients, hunters, and pregnant women would increase our understanding of transmission mechanisms and health outcomes among these high-risk groups. Lastly, developing continuing medical education programs to educate physicians on both diagnosis and treatment will decrease morbidity and mortality from this potentially fatal disease. As our literature review highlights, Chagas disease has consistently been reported in our state, Texas, since the 1930s. Collectively, we must mandate a change to focus attention on this important health disparity causing significant morbidity and mortality in the US.

**Top Five Papers**

1. Sarkar S, Strutuz SE, Frank DM, Rivaldi CL, Sissel B, Sanchez-Cordero V. Chagas disease risk in Texas. PLoS Negl Trop Dis. 2010. 4(10): e836
2. Hotez PJ, Dumonteil E, Betancourt Cravioto M, Bottazzi ME, Tapia-Conyer R, Meymandi S, Karunakara U, Ribeiro I, Cohen RM, Pecoul B. An unfolding tragedy of Chagas disease in North America. 2013. 7(10): e2300
3. Kjos SA, Snowden KF, Olson JK. Biogeography and Trypanosoma cruzi infection prevalence of Chagas disease vectors in Texas, USA. Vector Borne Zoonotic Dis. 2009. 9(1): 41–50
4. Bern C, Kjos S, Yabsley MJ, Montgomery SP. Trypanosoma cruzi and Chagas’ disease in the United States. Clin Microbiol Rev. 2011. 24(4): 655–681
5. Cantey PT, Stramer SL. Townsend RL, Kamel H, Ofafa K, Todd CW, Currier M, Hand S, Varnado W, Dotson E, Hall C, Jett PL, Montgomery SP. The United States Trypanosoma cruzi infection study: evidence for vector-borne transmission of the parasite that causes Chagas disease among United States blood donors. 2012. 52(9): 1922–1930

**Key Learning Points**

- Human exposure to *T*. *cruzi* from triatomine bites date back to 1935 in Texas.
- Disease burden from Chagas disease has been consistently documented over the past 70 years.
- Future public health interventions should focus on high-risk populations in the southern US, where locally acquired infection is likely.
